# Infant Growth after Preterm Birth and Mental Health in Young Adulthood

**DOI:** 10.1371/journal.pone.0137092

**Published:** 2015-09-01

**Authors:** Sara Sammallahti, Marius Lahti, Riikka Pyhälä, Jari Lahti, Anu-Katriina Pesonen, Kati Heinonen, Petteri Hovi, Johan G. Eriksson, Sonja Strang-Karlsson, Anna-Liisa Järvenpää, Sture Andersson, Eero Kajantie, Katri Räikkönen

**Affiliations:** 1 Institute of Behavioural Sciences, University of Helsinki, Helsinki, Finland; 2 National Institute for Health and Welfare, Helsinki, Finland; 3 Children’s Hospital, Helsinki University Central Hospital and University of Helsinki, Helsinki, Finland; 4 Folkhälsan Research Centre, Helsinki, Finland; 5 Department of General Practice and Primary Health Care, University of Helsinki, Helsinki, Finland; 6 Vasa Central Hospital, Vasa, Finland; 7 Unit of General Practice, Helsinki University Central Hospital, Helsinki, Finland; 8 Department of Obstetrics and Gynaecology, Oulu University Hospital and University of Oulu, Oulu, Finland; The Ohio State Unversity, UNITED STATES

## Abstract

**Objectives:**

Faster growth after preterm birth benefits long-term cognitive functioning. Whether these benefits extend to mental health remains largely unknown. We examined if faster growth in infancy is associated with better self-reported mental health in young adults born preterm at very low birth weight (VLBW) (<1500g).

**Study Design:**

As young adults, participants of the Helsinki Study of Very Low Birth Weight Adults self-reported symptoms of depression and attention deficit/hyperactivity disorder (ADHD) (n = 157) and other psychiatric problems (n = 104). As main predictors of mental health outcomes in linear regression models, we used infant weight, length, and head circumference at birth, term, and 12 months of corrected age, and growth between these time points. Growth data were collected from records and measures at term and at 12 months of corrected age were interpolated. Additionally, we examined the moderating effects of intrauterine growth restriction.

**Results:**

Size at birth, term, or 12 months of corrected age, or growth between these time points were not associated with mental health outcomes (p-values >0.05). Intrauterine growth restriction did not systematically moderate any associations.

**Conclusions:**

Despite the high variability in early growth of VLBW infants, the previously described association between slow growth in infancy and poorer cognitive functioning in later life is not reflected in symptoms of depression, ADHD, and other psychiatric problems. This suggests that the development of cognitive and psychiatric problems may have dissimilar critical periods in VLBW infants.

## Introduction

Decreasing the burden of preterm birth (<37 gestational weeks) has been deemed a public health priority[1] in developed countries, where every 10^th^ birth is preterm: not only does it represent the current leading cause of perinatal mortality[2], but it also poses a risk for poorer long-term cognitive[3–6] and social and emotional[7–9] functioning, and increases the risk of clinical and sub-clinical mental disorders[10–14]. Particularly those preterm individuals who have experienced restricted growth *in utero* may be at risk of poorer mental health[11,12,15], including internalizing problems[9], depression[16], and symptoms of attention deficit/hyperactivity disorder (ADHD)[17,18].

However, many individuals born preterm do not suffer from poorer long-term cognitive functioning and mental health. These differences may arise from factors that both underlie and result from being born too early, including the degree of immaturity, variations in intrauterine growth, maternal pregnancy disorders, and immaturity-associated illnesses and complications. Another factor behind these differences may be postnatal growth. A scant literature suggests that faster growth in infancy and in childhood may protect some individuals from risks associated with preterm birth. The literature on long-term consequences has thus far mainly concentrated on benefits that faster growth may offer for cognitive functioning[19–22]. We are aware of only three studies to date that have tested associations specifically between growth after preterm birth and mental health outcomes. One study reported that growth in head circumference during the first two years after preterm birth at extremely low birth weight (<1000g) was not associated with parent-/teacher-reported ADHD symptoms in childhood[23]. Another study found that weight gain from preterm birth to 4–7 years was not consistently associated with parent-rated internalizing or externalizing problems in childhood[24]. In contrast, we have recently reported that faster growth from birth to term, but not from term to 12 months of corrected age (CA) after preterm birth at very low birth weight (VLBW) (<1500g) was associated with lower self-reported autism-spectrum traits in young adulthood[25].

We now extend these studies by investigating if faster growth in weight, length, and head circumference from birth to term and thereafter to 12 months of CA in individuals born preterm at VLBW predicts better self-reported mental health, including lower depressive and ADHD symptoms and other psychiatric problems in young adulthood. Additionally, we compare the effects of growth in individuals who were born small- (SGA) (≤-2 standard deviations [SD] according to Finnish growth charts) or appropriate for gestational age (AGA) (>-2SD).

## Methods

### Participants

The original *Helsinki Study of Very Low Birth Weight Adults* cohort consisted of 335 VLBW infants born consecutively between January 1978 and December 1985 and discharged alive (survival rate 70.7%) from the Neonatal Intensive Care Unit of Children’s Hospital at Helsinki University Central Hospital in Finland. The cohort has been described elsewhere.[26] In 2004–2005, 255 individuals residing in the greater Helsinki area were invited to the first clinical follow-up visit[16,18]: 166 participated and were invited to the second follow-up visit[22,27,28] in 2007–2008. During the first and second visit, 164 and 108 participants, respectively, completed the mental health questionnaires. We excluded three participants who reported a developmental disability and four participants with no growth data available, which resulted in 158 VLBW participants in total, of whom 157 and 104 had available data on mental health outcomes on the first and second visit, respectively.

### Ethics statement

All participants gave their written informed consent as adults, and the Ethics Committee for Children and Adolescents’ Diseases and Psychiatry at the Helsinki University Central Hospital approved the study protocol. In order to protect the privacy of the cohort members, the data were anonymized during collection and provided to the researchers of the current study in a format where official personal identification numbers and other personal information such as names and addresses had been removed, and participants could only be identified using study identification codes.

### Growth measures

Weight, length, and head circumference measurements came from hospital and child welfare clinic records. To obtain measures at *term* (40+0 weeks+days postmenstrual age), we interpolated between true measurements, provided a measurement had been made within 28 days. Median time period between term and closest true measurement point was 1 day for weight, 5 days for length, and 4 days for head circumference. We interpolated size at *12 months* (52 weeks) *CA* if a measurement had been made within 42 days, allowing a wider range to increase sample size. Median time period between 12 months CA and closest true measurement point was 14 days for weight, and 15 days for length and head circumference.

We converted size at birth and at term into *z* scores by sex and age according to Finnish charts[29]. Finnish infant growth charts from that time[30,31] provide *z* scores for length and head circumference, and a percentage score of current weight in relation to expected weight for sex and CA. Therefore, we converted length and head circumference at 12 months CA into standardized *z* scores by sex and age, whereas weight at 12 months was first converted into percentage scores for sex and age and thereafter, to facilitate comparison of effect sizes, into *z* scores within the VLBW cohort.

### Outcome measures

During the first clinical visit, the participants completed the Beck Depression Inventory (BDI)[32] and the Center for Epidemiological Studies Depression Scale (CES-D)[33], which measure the severity and frequency of depressive symptoms, respectively. They also completed the Adult Problem Questionnaire (APQ)[34], which measures behavioral symptoms of ADHD. During the second clinical visit, the participants completed the Achenbach System of Empirically Based Assessment Adult Self Report (ASR)[35]. This questionnaire yields a Total Problems score reflecting overall psychosocial adjustment, and two subscores, the Internalizing Problems subscore reflecting symptoms of anxiety, depression, withdrawal, and somatic complaints, and the Externalizing Problems subscore reflecting delinquent and aggressive behavior symptoms. The mental health questionnaires used in this study have been used extensively in epidemiology and have good psychometric properties [34–36]. On all scales, a higher score reflects a higher number or more frequent/severe symptoms.

### Covariates and confounders

From medical records, we extracted sex (male/female), gestational age (weeks), date of birth for calculating age during the visit (years), time period between closest true measurement point and term /12 months CA (days), self-reported maternal smoking during pregnancy (yes/no), and neonatal complications/illnesses (septicemia, bronchopulmonary dysplasia, indomethacin treatment, surgery due to patent ductus arteriosus, and blood exchange transfusion due to hyperbilirubinemia [each yes/no]; duration of ventilator treatment [days]; and intraventricular hemorrhage [grade]). 47 participants lacked data on intraventricular hemorrhage, and 10 participants lacked data on maternal smoking during pregnancy, and were considered separates groups when dummy coding the variables. None of the participants were diagnosed with necrotizing enterocolitis. During the first clinical visit, participants reported diagnosed neurosensory impairments (cerebral palsy/blindness; none reported severe hearing impairment) and highest education of either parent (basic /secondary /lower tertiary /upper tertiary).

### Statistical analyses

As main outcomes in linear regression models, we used BDI and CES-D (after logarithmic transformation to attain normality), and APQ sumscores, and ASR Total Problems score and Internalizing and Externalizing Problems subscores (after square root transformation to attain normality). We standardized these outcomes by sex within the sample (mean = 0, SD = 1) to facilitate interpretation of effect sizes.

As main predictors of mental health outcomes, we used infant growth in weight, length, and head circumference from a) birth to term and b) from term to 12 months CA. We used standardized residual change scores from linear regression models where a) body size z scores at term were regressed on the corresponding measure at birth and b) body size z scores at 12 months CA were regressed on the corresponding measure at term, creating uncorrelated residuals that reflect growth conditional on previous history.[37,38] In supplementary analyses, we used body size z scores at birth, term, and 12 months CA to predict outcome variables. We considered two-tailed p-values <0.05 significant.

To control for covariates and confounders, we included gestational age at birth, sex, age during the visit in question, time period between closest true measurement point and term (and 12 months CA, when analyzing growth after term), and parental education (as a proxy of socio-economic background) in all analyses. In additional models, we further adjusted for neonatal complications/illnesses and maternal smoking during pregnancy, and excluded those with cerebral palsy (n = 13) and blindness (n = 2).

We first performed the analyses in the whole VLBW group. We then tested if the effects of growth on mental health varied by SGA/AGA status. We did this by including the interaction term ‘SGA vs. AGA status for weight /length /head circumference x corresponding growth measure’ into the regression equation followed by main effects. All participants were <+2SD in birth weight and head circumference. The two participants scoring >+2SD in birth length were classified as AGA for length.

## Results

### Participant characteristics and attrition

Data on participants’ growth in infancy and on neonatal and adult characteristics and mental health outcomes are presented in Table 1.

**Table 1:** Growth and infant and young adult characteristics of individuals born preterm at very low birth weight (<1500g).

	Participants of the 1^st^ clinical visit 2004–2005*		Participants of the 2^nd^ clinical visit 2007–2008*	
	M (SD)	N	M (SD)	N
**Characteristics at birth**				
Weight, kg	1.1 (0.2)	157	1.1 (0.2)	104
Length, cm	37 (2.4)	155	37 (2.5)	102
Head circumference, cm	26 (2.0)	155	26 (2.1)	101
SGA for weight: birth weight ≤-2 SD, n (%)	51 (32)	157	39 (38)	104
SGA for length: birth length ≤-2 SD, n (%)	49 (32)	155	30 (29)	102
SGA for head circumference: birth head circumference ≤-2 SD, n (%)	35 (23)	155	23 (23)	101
Extremely low birth weight: <1000 grams, n (%)	47 (30)	157	28 (27)	104
Gestational age, weeks	29 (2.2)	157	29 (2.4)	104
Very preterm: gestational age <32 weeks, n (%)	139 (89)	157	88 (85)	104
Extremely preterm: gestational age <28 weeks, n (%)	40 (25)	157	29 (28)	104
Mother reported smoking during pregnancy, yes, n (%)	28 (19)	147	16 (16)	97
Sex, male, n (%)	66 (42)	157	45 (43)	104
**Neonatal complications and illnesses**				
Duration of ventilator treatment, median days (25th to 75th percentile)	4.5 (0 to 14)	154	4.0 (0 to 15)	101
Septicemia, yes, n (%)	12 (8)	154	9 (9)	101
Bronchopulmonary dysplasia, yes, n (%)	29 (19)	152	25 (25)	100
Received indomethacin, yes, n (%)	44 (28)	155	33 (32)	102
Surgery due to patent ductus arteriosus, yes, n (%)	8 (5)	155	8 (8)	102
Blood exchange transfusion due to hyperbilirubinemia, yes, n (%)	25 (16)	155	15 (15)	102
Intraventricular hemorrhage, n (%)		111		77
none	90 (81)		65 (84)	
grade I or II	16 (14)		9 (12)	
grade III or IV	5 (5)		3 (4)	
**Characteristics at term**				
Weight, kg	2.5 (0.5)	157	2.5 (0.5)	104
Length, cm	46 (2.6)	150	46 (2.7)	101
Head circumference, cm	34 (1.9)	149	34 (2.0)	99
**Growth from birth to term**				
Weight change, kg	1.4 (0.4)	157	1.4 (0.5)	104
Length change, cm	9.0 (2.6)	148	9.0 (2.9)	99
Head circumference change, cm	7.6 (2.1)	147	7.5 (2.2)	96
**Characteristics at 12 months of corrected age**				
Weight, kg	8.5 (1.1)	131	8.6 (1.1)	87
Length, cm	73 (2.8)	127	73 (2.8)	84
Head circumference, cm	46 (1.4)	98	46 (1.4)	64
**Growth from term to 12 months of corrected age**				
Weight change, kg	6.0 (1.0)	131	6.0 (1.0)	87
Length change, cm	27 (2.7)	120	27 (2.6)	81
Head circumference change, cm	12 (1.5)	93	12 (1.6)	61
**Characteristics in adulthood**				
Age during clinical visit, years	22 (2.1)	157	25 (2.2)	104
Higher education of a parent, n (%)		157		104
basic/primary or less	17 (11)		11 (11)	
upper secondary	34 (22)		21 (20)	
lower tertiary	62 (39)		39 (38)	
upper tertiary	44 (28)		33 (32)	
**Mental health outcomes**				
Adult Problem Questionnaire sum score	38 (18)	157		
Beck Depression Inventory sum score	4.5 (5.4)	157		
Center for Epidemiologic Studies Depression Scale sum score	9.5 (7.5)	157		
ASEBA Adult Self Report Total Problems T-score			49 (10)	104
Internalizing Problems T-score			52 (13)	104
Externalizing Problems T-score			48 (9.7)	104

Abbreviations: AGA—appropriate for gestational age; CA—age corrected for prematurity; cm—centimeters, kg—kilograms, M—mean; N—number of participants for whom data were available; n—number of participants; SD—standard deviation; SGA—small for gestational age

* 158 VLBW individuals in total had data available on growth in infancy and adult mental health (after excluding those with developmental disability), and were thus included in the study. 157 participants had mental health data available from the 1^st^ visit, 104 had data available from the 2^nd^ visit, and 103 had data available from both visits.

Sample size varied according to growth data availability (Table 1). We found no differences in mental health questionnaire scores between those with and without growth data available in weight, length, and head circumference from birth to term or from term to 12 months CA (p-values >0.12). Those with weight growth data from birth to term (n = 158) did not differ in any available measures of body size or covariate / confounder data (p-values >0.12) from those without these data (n = 4), either. Those with length growth data from birth to term (n = 149) were less likely to have mothers who smoked during pregnancy (17% vs. 42%, p = 0.04) than those without these data (n = 13). Those with head circumference growth data from birth to term (n = 147) less often reported lower tertiary level as highest parental education (37% vs. 73%, p = 0.01) than those without these data (n = 15). Those with weight growth data from term to 12 months CA (n = 132) were more likely to be women (61% vs. 40%, p = 0.03) and more often had undergone blood exchange transfusion (19% vs. 3%, p = 0.04) than those without these data (n = 30). Those with length growth data from term to 12 months CA (n = 121) were also more likely to be women (64% vs. 39%, p = 0.01) and more often had undergone blood exchange transfusion (21% vs. 3%, p = 0.01) than those without these data (n = 41). Those with head circumference growth data from term to 12 months CA (n = 94) had higher SD scores for birth weight (mean [M] = -1.0 vs. M = -1.5, p = 0.04), birth length (M = -1.0 vs. M = -1.6, p = 0.04), and term length (M = -2.3 vs. M = -2.7, p = 0.05) than those without these data (n = 68).

### Infant growth and adult mental health in the VLBW group

**Fig 1:**
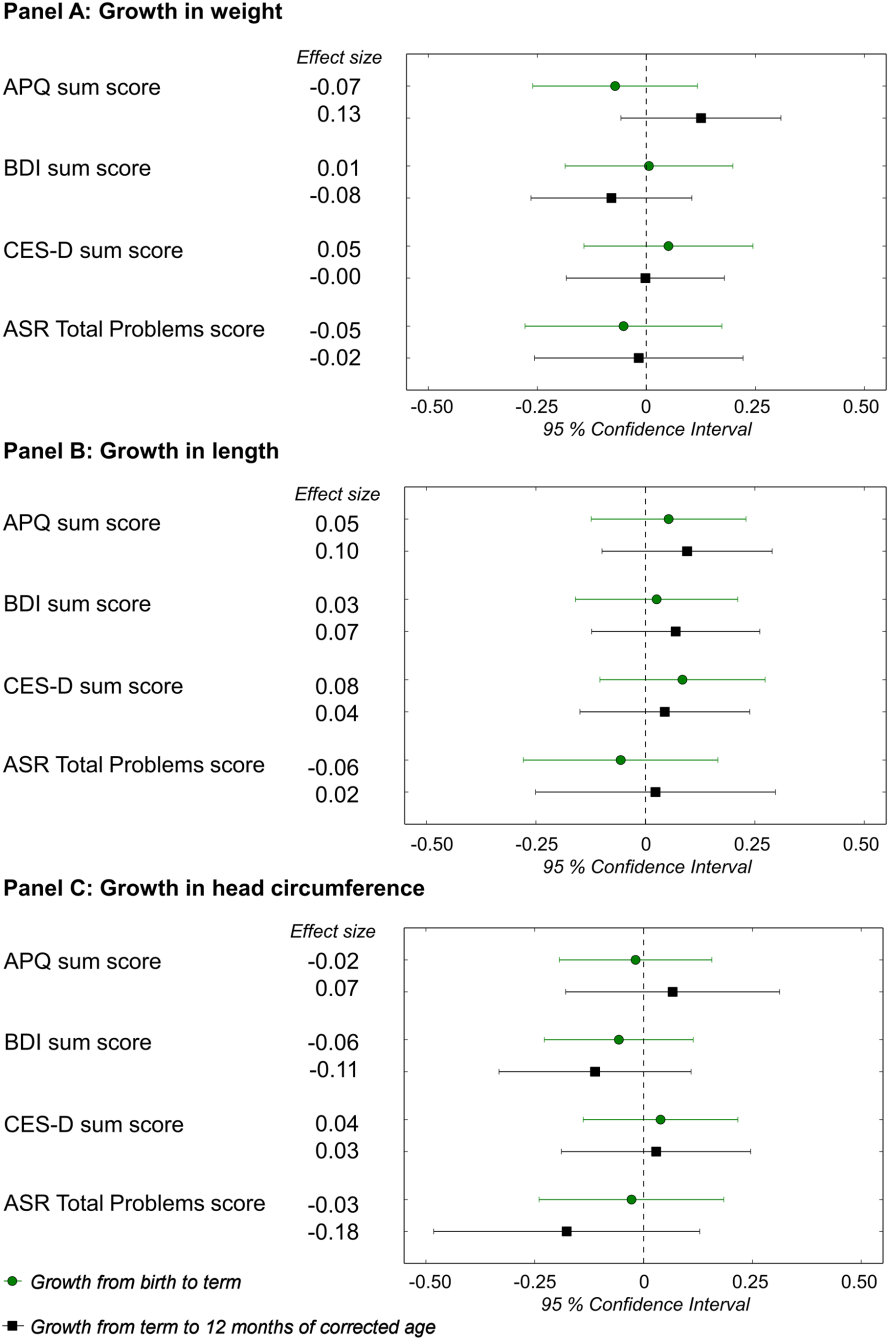
Growth in infancy and mental health questionnaire sumscores in very low birth weight adults. Change in mental health questionnaire sumscores (in SD units) in young adulthood per one SD faster growth from birth to term, and from term to 12 months of corrected age, in individuals with very low birth weight (<1500g). We adjusted for gestational age at birth, sex, age at completing questionnaire, highest education of a parent, and time period between closest true measurement point and term (and 12 months CA, when analyzing growth after term). Outcomes were standardized within the study group. Terms and abbreviations: APQ—Adult Problem Questionnaire, reflecting symptoms of attention deficit / hyperactivity disorder; BDI—Beck Depression Inventory; CES-D—Center for Epidemiologic Studies Depression scale; ASR—ASEBA Adult Self Report; SD—standard deviation; Effect size—standard deviation change in questionnaire score.

Firstly, we tested if *growth* from birth to term and from term to 12 months CA were associated with adult mental health outcomes. Fig 1 presents the main findings: associations between growth and APQ, BDI, CES-D, and ASR Total Problems sumscores. Additional results concerning ASR Internalizing and Externalizing subscales are shown in S1 Table. These associations were not statistically significant (Fig 1, S1 Table). The results remained similar after adjusting for neonatal complications/illnesses or maternal smoking during pregnancy, or excluding participants with neurosensory impairments.

We then tested if *body size* at birth, at term or at 12 months CA (S2 Table) were associated with adult mental health outcomes. These associations were not significant either (S2 Table), except when we made adjustments for neonatal complications/illnesses or maternal smoking during pregnancy, larger head circumference at term was associated with a lower Internalizing Problems subscore (-0.14 SD units per 1SD larger head circumference, 95% Confidence Interval [CI] -0.26 to -0.02, p = 0.02), and when we excluded participants with neurosensory impairments, larger head circumference at 12 months CA was associated with a lower Total Problems score (-0.33 SD units, 95% CI -0.66 to -0.01, p = 0.04) and Externalizing Problems subscore (-0.31 SD units, 95% CI -0.62 to -0.00, p = 0.05).

### Effects of growth in SGA and AGA subgroups

The effects of growth on mental health outcomes did not differ according to SGA/AGA status for weight or length (p-values>0.05 for small/appropriate birth weight for gestational age x weight growth interactions, and for small/appropriate birth length for gestational age x length growth interactions) (S3 Table). Faster growth in head circumference from birth to term was associated with higher depressive symptoms scores in individuals born SGA for head circumference (CES-D scores increased by 0.61 SD units per 1SD faster growth [95% CI 0.13 to 1.09, p = 0.02]) but not in individuals born AGA for head circumference (p = 0.16) (p-value for small/appropriate head circumference for gestational age x head circumference growth from birth to term interaction = 0.001). The same interaction was significant when examining depressive symptoms measured by BDI (p = 0.047), but growth in head circumference from birth to term was not significantly associated with BDI scores in either SGA or AGA group (p-values>0.05) (S3 Table).

## Discussion

We show that self-reported symptoms of depression, ADHD, and other psychiatric problems in young adulthood were not associated with growth from birth to term or from term to 12 months CA after preterm birth at VLBW. We conducted 36 analyses examining associations between growth in weight, length, and head circumference during two time periods, and mental health questionnaire scores, and 54 analyses examining associations between body size at birth, at term, and at 12 months CA, and mental health questionnaire scores, and found no statistically significant effects. Further, we examined 36 interactions with intrauterine growth restriction, and found only two statistically significant interactions. In sub-analyses following one of them, we found statistically significant main effects: faster growth in head circumference from birth to term in those born SGA was related to higher depressive symptoms in young adulthood.

Our findings are contrary to what we expected based on previous literature, which has however concentrated on cognitive functioning as the long-term outcome. These studies have shown that faster growth after preterm birth may provide long-term benefits for intelligence and executive functioning[19–22]. Our recent findings in a subsample of this Helsinki Study of Very Low Birth Weight Adults cohort have shown that faster growth from birth to term, but not from term to 12 months of CA, particularly in head circumference, was associated with better general neurocognitive abilities, executive functioning, and visual memory in young adulthood[22]. We also recently demonstrated that among these VLBW participants, faster growth in weight, length, and head circumference from birth to term, but not from term to 12 months of CA, was associated with lower self-reported autism-spectrum traits[25]. Although cognitive functioning and mental health are often correlated, the conclusion from this study of individuals born preterm at VLBW with high variability in early growth is that the underlying mechanisms of resilience and pathways to better cognitive functioning and mental health seem at least partly different. In line, it has recently been suggested that also the underlying mechanisms responsible for the associations between preterm birth and morbidity may be outcome-specific[14,39].

Our findings are in agreement with the two previous studies that we are aware of, which reported that growth in head circumference during the first two years of life after preterm birth at extremely low birth weight was not associated with parent- or teacher-reported ADHD symptoms in childhood[23], and weight gain from preterm birth to 4–7 years was not associated with parent-rated behavioral symptoms in childhood[24]. Since publication bias favors reporting of false positive findings, null findings from our and previous studies are particularly important for understanding the factors and pathways that underlie individual variations in cognitive and mental health outcomes after preterm birth. Clearly, further studies that either confirm or refute these null findings are warranted.

We did not find that the effects of growth on mental health among the preterm VLBW group would vary systematically by intrauterine growth restriction, as reflected by AGA/SGA status, either, and cannot rule out that the few significant associations reflect type 1 error. This finding is in agreement with our own previous reports on cognitive functioning[22] and autism-spectrum traits[25]: in these studies, we showed that faster growth after preterm birth at VLBW benefitted those born SGA or AGA equally well.

In addition to neurocognitive outcomes, earlier studies have shown increased cardiometabolic risk factors such as higher blood pressure and impaired glucose regulation in adults born preterm[40]. In relation to early growth, some authors have suggested a “tradeoff”, meaning that faster postnatal growth in preterm infants would improve neurodevelopment, but also increase risk factors for later cardiovascular disease[41]. Others have suggested that promoting faster growth immediately after preterm birth may benefit both brain development and cardiometabolic health, whereas rapid weight gain after term may have harmful cardiometabolic consequences[42,43]. Among this cohort of VLBW adults, we have previously shown that those who gained weight more rapidly between birth and term had a favorable cardiometabolic profile, including lower blood pressure[44], higher brachial artery flow-mediated dilatation indicating better endothelial function[45], and smaller carotid artery intima-media thickness[45]. However, in the relatively small group of participants who had suffered from intrauterine growth restriction, those with more rapid weight gain from birth to term had higher fasting insulin concentrations[26]. Taken together, the studies conducted in this cohort of VLBW adults who, by the standards of today, grew on average slowly, seem to indicate that faster growth immediately after preterm birth may benefit some, but not all long-term physical and mental health outcomes.

Strengths of this study include the longitudinal study design, extensive early life and adult mental health data, and the use of well-validated mental health questionnaires. Limitations include sample size: while these 157 and 104 adults provide insight into the associations between early growth and mental health in the VLBW population as a whole, postnatal growth may be associated with adult mental health in some VLBW individuals (e.g. those born to mothers who had hypertensive pregnancy disorders, which complicate approximately 4–10% of all pregnancies[46]) and not others, yet larger samples would be needed to show these group-specific effects. Since small sample size and multiple statistical testing may introduce type 1 and type 2 errors, especially the findings in the SGA group should be interpreted with caution. Also, follow-up attrition may have caused over-representation of healthier participants, even though we did not find that mental health would have differed between those who had and those who did not have data available on early growth. Self-reported symptomatology and problems of mental health are screening tools, and do not provide diagnoses. Therefore, the findings do not generalize to groups with diagnosed mental disorders. We also stress that we examined growth after preterm birth, with a particular interest in the time period the term-born infant spends *in utero*: associations between growth and mental health in term-born populations, and conclusions about potential differences between preterm and term-born individuals were beyond the scope of our study. Finally, the participants in this study were born between 1978 and 1985, and thus the findings may not be representative of preterm infants born today in high-income settings, whose postnatal care and nutrition have much improved, and who grow on average substantially faster than the infants we have studied. This is an unavoidable limitation, when examining adult outcomes. This can also be considered a study strength, as the group showed high variability in early growth, and thus provided a unique opportunity for studying variation in early growth.

In conclusion, we show that growth in infancy after preterm birth at VLBW seems largely unrelated to self-reported symptoms of depression, ADHD, and other psychiatric problems in young adulthood. We also show that intrauterine growth restriction, as reflected by SGA and AGA status, did not systematically moderate effects of growth. Further studies are clearly warranted that unravel factors explaining why some individuals born preterm at VLBW are rendered vulnerable to mental disorders, while others remain resilient.

## Supporting Information

S1 Table Growth in infancy and ASR Internalizing and Externalizing subscale scores. Change in ASR Internalizing and Externalizing subscale scores (in SD units) per one SD faster growth in weight (Panel A), length (Panel B), and head circumference (Panel C) during two time periods in infancy: birth to term, and term to 12 months CA, in individuals born at very low birth weight (<1500g).
(PDF)

S2 Table Size in infancy and mental health questionnaire scores in very low birth weight adults. Change in mental health questionnaire scores (in SD units) in young adulthood per one unit difference in relative weight (Panel A), length (Panel B), and head circumference (Panel C) at birth, at term equivalent age, and at 12 months of corrected age in individuals with very low birth weight (<1500g).
(PDF)

S3 Table Growth in infancy and adult mental health: comparisons between SGA and AGA preterm individuals. Change in adult mental health questionnaire scores (in SD units) per one SD faster growth in weight (Panel A), length (Panel B), and head circumference (Panel C) during two time periods in infancy: birth to term, and term to 12 months CA. Results are shown separately for small-for-gestational-age (SGA) and appropriate-for-gestational-age (AGA) individuals, who were all born preterm at very low birth weight (<1500g).
(PDF)
