# Supplementary material for: Infant Growth after Preterm Birth and Mental Health in Young Adulthood
Source: PLoS One. 2015 Sep 1;10(9):e0137092. doi: 10.1371/journal.pone.0137092 (PMC4556664; doi:10.1371/journal.pone.0137092)
Supplement: S1 Table — Change in ASR Internalizing and Externalizing subscale scores (in SD units) per one SD faster growth in weight (Panel A), length (Panel B), and head circumference (Panel C) during two time periods in infancy: birth to term, and term to 12 months CA, in individuals born at very low birth weight (<1500g). (PDF) [file pone.0137092.s001.pdf]

S1 Table. Growth in infancy and ASR Internalizing and Externalizing subscale scores. Change in ASR Internalizing and Externalizing subscale scores (in SD units) per one SD faster growth in weight (Panel A), length (Panel B), and head circumference (Panel C) during two time periods in infancy: birth to term, and term to 12 months CA, in individuals born at very low birth weight (<1500g).

We adjusted for gestational age at birth, sex, age at completing questionnaire, highest education of a parent, and time period between closest true measurement point and term (and 12 months CA, when analyzing growth after term). ASEBA Adult Self Report T-scores were standardized within the study group.

Terms and abbreviations: ASR – ASEBA Adult Self Report; CI – Confidence Interval; Effect size – standard deviation change in questionnaire score; SD – standard deviation

Panel A: Growth in weight

Birth to term		
ASR subscale score	*Effect size*	*95% CI*
Internalizing	-0.09	-0.32 to 0.13
Externalizing	-0.03	-0.26 to 0.19
Term to 12 months CA		
ASR subscale score	*Effect size*	*95% CI*
Internalizing	-0.09	-0.32 to 0.13
Externalizing	0.13	-0.11 to 0.36

Panel B: Growth in length

Birth to term		
ASR subscale score	*Effect size*	*95% CI*
Internalizing	-0.11	-0.33 to 0.11
Externalizing	-0.07	-0.28 to 0.15
Term to 12 months CA		
ASR subscale score	*Effect size*	*95% CI*
Internalizing	-0.05	-0.31 to 0.21
Externalizing	0.10	-0.18 to 0.37

Panel C: Growth in head circumference

Birth to term		
ASR subscale score	*Effect size*	*95% CI*
Internalizing	-0.10	-0.31 to 0.11
Externalizing	0.05	-0.16 to 0.27
Term to 12 months CA		
ASR subscale score	*Effect size*	*95% CI*
Internalizing	-0.14	-0.42 to 0.15
Externalizing	-0.19	-0.49 to 0.10
