# Supplementary material for: Infant Growth after Preterm Birth and Mental Health in Young Adulthood
Source: PLoS One. 2015 Sep 1;10(9):e0137092. doi: 10.1371/journal.pone.0137092 (PMC4556664; doi:10.1371/journal.pone.0137092)
Supplement: S2 Table — Change in mental health questionnaire scores (in SD units) in young adulthood per one unit difference in relative weight (Panel A), length (Panel B), and head circumference (Panel C) at birth, at term equivalent age, and at 12 months of corrected age in individuals with very low birth weight (<1500g). (PDF) [file pone.0137092.s002.pdf]

**S2 Table. Size in infancy and mental health questionnaire scores in very low birth weight adults.** Change in mental health questionnaire scores (in SD units) in young adulthood per one unit difference in relative weight (Panel A), length (Panel B), and head circumference (Panel C) at birth, at term equivalent age, and at 12 months of corrected age in individuals with very low birth weight (<1500g).

We adjusted for gestational age at birth, sex, time period between closest true measurement point and term / 12 months CA, age at completing questionnaire, and highest education of a parent. Weight at 12 months CA was converted into percentages of expected weight according to national growth charts and then converted into z scores within the VLBW sample; all other measurements were standardized according to national growth charts. Outcomes were standardized within the study group.

Terms and abbreviations: APQ – Adult Problem Questionnaire sumscore, reflecting symptoms of attention deficit / hyperactivity disorder; BDI – Beck Depression Inventory sumscore, reflecting the severity of depressive symptoms; CES-D – Center for Epidemiologic Studies Depression scale sumscore, reflecting the frequency of depressive symptoms; CI – Confidence Interval; ASR – ASEBA Adult Self Report T-scores, reflecting overall psychosocial adjustment (Total Problems), symptoms of anxiety, depression, withdrawal and somatic complaints (Internalizing) and delinquent and aggressive behavior symptoms (Externalizing); SD – standard deviation; SDS – Standard deviation score; Effect size - standard deviation change in questionnaire score

**Panel A: Weight SDS**

***Weight at birth***

| *Mental health outcome* | *Effect size* | *95% CI* |
|---|---|---|
| APQ | 0.00 | -0.14 to 0.15 |
| BDI | -0.08 | -0.23 to 0.07 |
| CES-D | -0.04 | -0.19 to 0.11 |
| ASR Total Problems | -0.03 | -0.21 to 0.16 |
| ASR Internalizing | -0.02 | -0.21 to 0.17 |
| ASR Externalizing | -0.07 | -0.25 to 0.12 |

***Weight at term***

| *Mental health outcome* | *Effect size* | *95% CI* |
|---|---|---|
| APQ | -0.05 | -0.20 to 0.11 |
| BDI | -0.06 | -0.21 to 0.10 |
| CES-D | 0.01 | -0.15 to 0.17 |
| ASR Total Problems | -0.06 | -0.25 to 0.14 |
| ASR Internalizing | -0.08 | -0.27 to 0.11 |
| ASR Externalizing | -0.07 | -0.26 to 0.12 |

***Weight at 12 months CA***

| *Mental health outcome* | *Effect size* | *95% CI* |
|---|---|---|
| APQ | 0.12 | -0.06 to 0.30 |
| BDI | -0.08 | -0.26 to 0.10 |
| CES-D | 0.03 | -0.15 to 0.21 |
| ASR Total Problems | -0.02 | -0.25 to 0.20 |
| ASR Internalizing | -0.09 | -0.30 to 0.13 |
| ASR Externalizing | 0.07 | -0.16 to 0.29 |

**Panel B: Length SDS**

***Length at birth***

| *Mental health outcome* | *Effect size* | *95% CI* |
|---|---|---|
| APQ | -0.05 | -0.16 to 0.06 |
| BDI | -0.03 | -0.14 to 0.09 |
| CES-D | -0.03 | -0.14 to 0.09 |
| ASR Total Problems | 0.04 | -0.11 to 0.20 |
| ASR Internalizing | 0.06 | -0.09 to 0.22 |
| ASR Externalizing | 0.04 | -0.11 to 0.19 |

***Length at term***

| *Mental health outcome* | *Effect size* | *95% CI* |
|---|---|---|
| APQ | -0.01 | -0.12 to 0.10 |
| BDI | 0.00 | -0.12 to 0.12 |
| CES-D | 0.02 | -0.10 to 0.14 |
| ASR Total Problems | -0.03 | -0.18 to 0.11 |
| ASR Internalizing | -0.04 | -0.19 to 0.11 |
| ASR Externalizing | -0.05 | -0.19 to 0.09 |

***Length at 12 months CA***

| *Mental health outcome* | *Effect size* | *95% CI* |
|---|---|---|
| APQ | 0.08 | -0.09 to 0.25 |
| BDI | 0.01 | -0.16 to 0.18 |
| CES-D | 0.06 | -0.10 to 0.23 |
| ASR Total Problems | 0.03 | -0.18 to 0.25 |
| ASR Internalizing | -0.03 | -0.23 to 0.17 |
| ASR Externalizing | 0.08 | -0.13 to 0.29 |

**Panel C: Head circumference SDS**

***Head circumference at birth***

| *Mental health outcome* | *Effect size* | *95% CI* |
|---|---|---|
| APQ | -0.04 | -0.17 to 0.09 |
| BDI | -0.10 | -0.23 to 0.03 |
| CES-D | -0.07 | -0.21 to 0.06 |
| ASR Total Problems | -0.03 | -0.19 to 0.14 |
| ASR Internalizing | -0.05 | -0.22 to 0.12 |
| ASR Externalizing | 0.00 | -0.17 to 0.16 |

***Head circumference at term***

| *Mental health outcome* | *Effect size* | *95% CI* |
|---|---|---|
| APQ | -0.01 | -0.09 to 0.07 |
| BDI | -0.04 | -0.12 to 0.04 |
| CES-D | 0.00 | -0.08 to 0.08 |
| ASR Total Problems | -0.04 | -0.13 to 0.06 |
| ASR Internalizing | -0.07 | -0.16 to 0.02 |
| ASR Externalizing | -0.02 | -0.11 to 0.08 |

***Head circumference at 12 months CA***

| *Mental health outcome* | *Effect size* | *95% CI* |
|---|---|---|
| APQ | -0.03 | -0.28 to 0.22 |
| BDI | -0.19 | -0.42 to 0.05 |
| CES-D | -0.06 | -0.28 to 0.16 |
| ASR Total Problems | -0.29 | -0.60 to 0.03 |
| ASR Internalizing | -0.24 | -0.54 to 0.06 |
| ASR Externalizing | -0.28 | -0.58 to 0.02 |
