# Supplementary material for: Infant Growth after Preterm Birth and Mental Health in Young Adulthood
Source: PLoS One. 2015 Sep 1;10(9):e0137092. doi: 10.1371/journal.pone.0137092 (PMC4556664; doi:10.1371/journal.pone.0137092)
Supplement: S3 Table — Change in adult mental health questionnaire scores (in SD units) per one SD faster growth in weight (Panel A), length (Panel B), and head circumference (Panel C) during two time periods in infancy: birth to term, and term to 12 months CA. Results are shown separately for small-for-gestational-age (SGA) and appropriate-for-gestational-age (AGA) individuals, who were all born preterm at very low birth weight (<1500g). (PDF) [file pone.0137092.s003.pdf]

S3 Table. Growth in infancy and adult mental health: comparisons between SGA and AGA preterm individuals. Change in adult mental health questionnaire scores (in SD units) per one SD faster growth in weight (Panel A), length (Panel B), and head circumference (Panel C) during two time periods in infancy: birth to term, and term to 12 months CA. Results are shown separately for small-for-gestational-age (SGA) and appropriate-for-gestational-age (AGA) individuals, who were all born preterm at very low birth weight (<1500g).

We adjusted for gestational age at birth, sex, age at completing questionnaire, highest education of a parent, and time period between closest true measurement point and term (and 12 months CA, when analyzing growth after term). Outcomes were standardized within the study group.

Terms and abbreviations: AGA – appropriate for gestational age, i.e. weight (panel A) / length (panel B) / head circumference (panel C) at birth >-2SD; APQ – Adult Problem Questionnaire sumscore, reflecting symptoms of attention deficit / hyperactivity disorder; ASR – Adult Self Report T-scores, reflecting overall psychosocial adjustment (Total Problems), symptoms of anxiety, depression, withdrawal and somatic complaints (Internalizing) and delinquent and aggressive behavior symptoms (Externalizing); BDI – Beck Depression Inventory sumscore; CA – corrected age; CES-D – Center for Epidemiological Studies Depression Scale sumscore; CI – Confidence Interval; Effect size - standard deviation change in questionnaire score; Interaction – p-value for the interaction ‘growth x AGA/SGA preterm status’ when predicting the mental health outcome in question; n – number of (SGA or AGA) participants for whom data were available; SD – standard deviation; SGA – small for gestational age, i.e. weight (panel A) / length (panel B) / head circumference (panel C) at birth ≤-2SD

Panel A: Growth in weight

Birth to term	*SGA*			*AGA*			*Interaction*
Mental health outcome	*Effect size*	*95% CI*	*n*	*Effect size*	*95% CI*	*n*	*p*
APQ	0.27	-0.17 to 0.71	51	-0.20	-0.41 to 0.02	106	0.40
BDI	0.18	-0.27 to 0.63	51	-0.07	-0.29 to 0.15	106	0.10
CES-D	0.32	-0.17 to 0.81	51	-0.04	-0.25 to 0.17	106	0.38
ASR Total Problems	-0.08	-0.51 to 0.35	39	-0.07	-0.38 to 0.24	65	0.71
ASR Internalizing	-0.15	-0.64 to 0.35	39	-0.03	-0.32 to 0.26	65	0.98
ASR Externalizing	-0.11	-0.49 to 0.26	39	-0.09	-0.39 to 0.22	65	0.90

Term to 12 months CA	*SGA*			*AGA*			*Interaction*
Mental health outcome	*Effect size*	*95% CI*	*n*	*Effect size*	*95% CI*	*n*	*p*
APQ	-0.09	-0.52 to 0.34	40	0.09	-0.12 to 0.30	91	0.96
BDI	0.01	-0.43 to 0.45	40	-0.15	-0.37 to 0.07	91	0.36
CES-D	0.07	-0.39 to 0.53	40	-0.03	-0.23 to 0.18	91	0.72
ASR Total Problems	0.35	-0.07 to 0.78	32	-0.24	-0.60 to 0.12	55	0.06
ASR Internalizing	0.28	-0.18 to 0.74	32	-0.23	-0.56 to 0.10	55	0.10
ASR Externalizing	0.46	0.06 to 0.85	32	-0.11	-0.45 to 0.24	55	0.09

Panel B: Growth in length

Birth to term	*SGA*			*AGA*			*Interaction*
Mental health outcome	*Effect size*	*95% CI*	*n*	*Effect size*	*95% CI*	*n*	*p*
APQ	0.39	-0.08 to 0.86	46	-0.04	-0.23 to 0.15	102	0.41
BDI	0.13	-0.36 to 0.63	46	-0.03	-0.23 to 0.18	102	0.38
CES-D	0.56	0.07 to 1.06	46	-0.02	-0.23 to 0.18	102	0.10
ASR Total Problems	-0.05	-0.56 to 0.46	29	-0.09	-0.36 to 0.18	70	0.84
ASR Internalizing	0.06	-0.46 to 0.57	29	-0.16	-0.43 to 0.12	70	0.61
ASR Externalizing	-0.21	-0.66 to 0.25	29	-0.08	-0.34 to 0.17	70	0.89

Term to 12 months CA	*SGA*			*AGA*			*Interaction*
Mental health outcome	*Effect size*	*95% CI*	*n*	*Effect size*	*95% CI*	*n*	*p*
APQ	-0.09	-0.69 to 0.51	36	0.07	-0.13 to 0.26	82	0.58
BDI	0.25	-0.34 to 0.85	36	-0.01	-0.20 to 0.19	82	0.54
CES-D	0.00	-0.61 to 0.61	36	0.05	-0.15 to 0.24	82	0.52
ASR Total Problems	0.33	-0.44 to 1.11	24	-0.11	-0.46 to 0.24	55	0.70
ASR Internalizing	0.47	-0.28 to 1.22	24	-0.21	-0.53 to 0.11	55	0.47
ASR Externalizing	0.16	-0.61 to 0.92	24	0.06	-0.28 to 0.41	55	0.82

Panel C: Growth in head circumference

Birth to term	*SGA*			*AGA*			*Interaction*
Mental health outcome	*Effect size*	*95% CI*	*n*	*Effect size*	*95% CI*	*n*	*p*
APQ	0.21	-0.33 to 0.74	33	-0.11	-0.29 to 0.08	114	0.26
BDI	0.31	-0.12 to 0.74	33	-0.18	-0.37 to 0.01	114	0.05
CES-D	0.61	0.13 to 1.09	33	-0.14	-0.32 to 0.05	114	0.001
ASR Total Problems	0.20	-0.47 to 0.87	22	-0.05	-0.30 to 0.19	74	0.48
ASR Internalizing	0.21	-0.35 to 0.78	22	-0.12	-0.37 to 0.13	74	0.40
ASR Externalizing	0.20	-0.41 to 0.81	22	0.02	-0.23 to 0.27	74	0.52

Term to 12 months CA	*SGA*			*AGA*			*Interaction*
Mental health outcome	*Effect size*	*95% CI*	*n*	*Effect size*	*95% CI*	*n*	*p*
APQ	0.19	-0.52 to 0.91	22	0.10	-0.17 to 0.37	70	0.92
BDI	0.24	-0.30 to 0.77	22	-0.23	-0.49 to 0.04	70	0.20
CES-D	0.21	-0.36 to 0.79	22	-0.03	-0.28 to 0.22	70	0.51
ASR Total Problems	0.15	-0.61 to 0.91	16	-0.36	-0.79 to 0.07	43	0.21
ASR Internalizing	0.16	-0.47 to 0.79	16	-0.33	-0.74 to 0.08	43	0.20
ASR Externalizing	0.09	-0.62 to 0.80	16	-0.42	-0.83 to 0.00	43	0.15
